# The Tonsil Lymphocyte Landscape in Pediatric Tonsil Hyperplasia and Obstructive Sleep Apnea

**DOI:** 10.3389/fimmu.2021.674080

**Published:** 2021-10-22

**Authors:** Anna Carrasco, Isabella Sjölander, Aline Van Acker, Andy Dernstedt, Johan Fehrm, Mattias Forsell, Danielle Friberg, Jenny Mjösberg, Anna Rao

**Affiliations:** ^1^ Center for Infectious Medicine, Department of Medicine Huddinge, Karolinska Institutet, Karolinska University Hospital, Stockholm, Sweden; ^2^ Department of Surgical Sciences, Otorhinolaryngology-Head and Neck Surgery, Uppsala University, Uppsala, Sweden; ^3^ Department of Clinical Microbiology, Section of Infection and Immunology, Umeå University, Umeå, Sweden; ^4^ Department of Clinical Sciences, Intervention, and Technology, Karolinska Institutet, Stockholm, Sweden

**Keywords:** Tonsils, obstructive sleep apnea, T cells, Innate lymphoid cell (ILC), B cells

## Abstract

Tonsil hyperplasia is the most common cause of pediatric obstructive sleep apnea (OSA). Despite the growing knowledge in tissue immunology of tonsils, the immunopathology driving tonsil hyperplasia and OSA remains unknown. Here we used multi-parametric flow cytometry to analyze the composition and phenotype of tonsillar innate lymphoid cells (ILCs), T cells, and B cells from pediatric patients with OSA, who had previous polysomnography. Unbiased clustering analysis was used to delineate and compare lymphocyte heterogeneity between two patient groups: children with small tonsils and moderate OSA (n = 6) or large tonsils and very severe OSA (n = 13). We detected disturbed ILC and B cell proportions in patients with large tonsils, characterized by an increase in the frequency of naïve CD27^-^CD21^hi^ B cells and a relative reduction of ILCs. The enrichment of naïve B cells was not commensurate with elevated Ki67 expression, suggesting defective differentiation and/or migration rather than cellular proliferation to be the causative mechanism. Finally, yet importantly, we provide the flow cytometry data to be used as a resource for additional translational studies aimed at investigating the immunological mechanisms of pediatric tonsil hyperplasia and OSA.

## Introduction

Pediatric obstructive sleep apnea (OSA) is a serious and common form of sleep disordered breathing in children characterized by repeated events of upper airway obstruction during sleep (1). OSA has been associated with serious complications, such as failure to thrive, cardiovascular disorders, hyperactivity, cognitive disturbances and lower quality of life (2–4). Hyperplasia of the adenoid and/or the palatine tonsils is the most common cause of OSA, which affects around 1% to 5% of all children between two to six years of age (5). Adenotonsillectomy is considered the first-line treatment for moderate to severe OSA and is one of the most common surgical procedures among children around the world, used since around 1000 B.C.E (6–8). While the immunological effects of adenotonsillectomy have been debated, two systematic reviews concluded that tonsillectomy does not have a negative impact on children’s humoral and cellular immunity (9, 10). However, adenotonsillectomy entails a considerable risk of postoperative hemorrhage and pain for the child. Therefore, further research on effective non-invasive treatments of tonsil hyperplasia is necessary, especially for children with severe OSA. Previous studies have suggested increased inflammation in OSA tonsils, including increased T-cell proliferation, elevated levels of pro-inflammatory cytokines (11), substance P (12) and upregulation of cysteinyl leukotrienes receptors (13). However, anti-inflammatory medications, such as nasal steroids and/or leukotriene receptor antagonists, have only shown short-term effects on mild OSA (14). These clinical observations warrant further investigation of the immunopathology of tonsil hyperplasia in children with OSA.

Palatine tonsils (further tonsils) is a mucosa-associated lymphoid tissue located in the upper airways, between the pillars in the pharynx (15). Tonsils represent the first site for immunologic response to inhaled and ingested pathogens together with the adenoid tissue and the lingual tonsils. Luminal antigens are taken up by specialized membranous (M) cells and transported to the lymphoid area of the tonsil, characterized by a high number of lymphoid follicles, containing differentiating B cells and follicular helper T cells (T_FH_) (16). More recently, the presence of helper innate lymphoid cells (ILCs) was described in human tonsillar tissue (17–19). ILCs are lymphocytes that lack re-arranged antigen-specific receptors and exert helper functions in response to environmental stimuli. Based on their phenotypical and functional profile helper ILCs are divided in four sub-groups: ILC1, ILC2, ILC3 and lymphoid-tissue inducer (LTi) cells (20). ILC1, ILC2 and ILC3 depend on the expression of the lineage transcription factors T-bet, GATA3 and RORγt, respectively and produce effector cytokines akin to T_H_1, T_H_2 and T_H_17/22 cells (21). LTi cells were shown to be involved in the formation of secondary lymphoid organs (SLOs) during mouse embryogenesis and, similar to ILC3, depend on the transcription factor RORγt (22). Although a phenotypically similar population of LTi-like cells can be detected in human fetal tissue and SLOs (23, 24), human LTi-like cells remain poorly characterized.

Here we investigated how the adaptive and innate lymphocyte populations differ in tonsillar tissue of children divided into two categories: small tonsils and moderate OSA, compared to large tonsils and very severe OSA. Using multi-color flow cytometry the composition and phenotype of ILCs, T cells and B cells were analyzed, addressing their differentiation and activation state. Uniform manifold approximation and projection (UMAP) (25) and PhenoGraph (26) algorithms separated cell populations based on lineage and maturation states, revealing additional layers of heterogeneity. Using these methods, an increased frequency of CD27^-^CD21^hi^ naïve B cells in large tonsils from pediatric patients with very severe OSA was observed. This enrichment was not paralleled by increased Ki67 expression, suggesting defective differentiation and/or migratory mechanisms in OSA tonsils. Our findings generate new hypotheses to be further explored in additional studies of immune mechanisms, including innate and adaptive lymphocytes, in tonsil hyperplasia and OSA. The underlying raw data for this project is provided as a resource for other researchers.

## Materials And Methods

### Study Design and Patient Samples

Human tonsils were obtained from children aged between two and five years who underwent tonsillectomies between 2014 to 2017 at the Otorhinolaryngological department (ORLD) Karolinska University Hospital Huddinge, Sweden. Written informed consent was obtained from the parents/guardians of the patients. Sample collection was approved by the Swedish Ethical Review Authority (Dnr 2014/1000-31/1 NCT02315911).

Tonsil hypertrophy was scored according to Brodsky grading (**Table 1**) (27). The group with tonsil hyperplasia (n=13) had significantly enlarged tonsils, size 4, in combination with very severe OSA, defined by an obstructive apnea–hypopnea index (OAHI) ≥ 30. Children with small tonsils (n=6) had tonsil size 2, with the exception of one patient (tonsil size 2.5). All six patients had an OAHI of 11 or less, which was termed “moderate” OSA. The exclusion criteria were the presence of craniofacial abnormality, neuromuscular disease, chromosomal abnormality, previous adenotonsillar surgery, bleeding disorder and cardiopulmonary disease. All 19 children had undergone polysomnography (PSG) overnight in a sleep laboratory at the ORLD to diagnose their degree of OSA before surgery. PSG measures sleep stages and respiratory functions using recordings of electroencephalogram, electro-oculogram, electromyogram, pulse, oronasal airflow, transcutaneous oxygen saturation, respiratory movements (abdomen and thorax), body position, and video and sound recordings. All PSGs were with the EMBLA technology (Flaga Medical, Iceland), and scored manually by the same registered polysomnography technologist according to the scoring rules of the American Academy of Sleep Medicine. The OAHI was calculated from the PSG scoring. There were no differences in age, sex distribution, weight or height between the groups. The children were all normal- or underweighted except for one overweight child, who also had large tonsils. No child was obese. Details of the patient cohort are provided in **Table 1**.

**Table 1:** Baseline characteristics of the patient cohort.

Parameter	Small	Large	p
	(n = 6)	(n = 13)	
**Age at operation, mean (SD), months**	38 (8)	34 (8)	0.4
**Sex, No. (%)**			
**Male**	2 (33)	8 (38)	
**Female**	4 (67)	5 (62)	
**Height, mean (SD), cm**	94 (7)^a^	92(5)	0.9
**Weight, mean (SD), kg**	14 (2)^a^	14 (3)	0.9
**BMI z-score, mean (SD)**	-0.7 (2.0)^a^	-0.4 (1.8)	0.6
**Tonsil size**^**b**^ **, median (IQR)**	2 (2–2.5)	4 (4–4)	<0.001
**OAHI, median (IQR), events/hour of sleep**	9.5 (7–11)	35 (33–36)	<0.001

ATE, adenotonsillectomy; OAHI, Obstructive Apnea-Hypopnea Index.

a One missing value in the group with small tonsils (n = 5).

b Tonsil size scored according to Brodsky (scored according to occlusion (%) of the oropharynx: 1 = 0–25%, 2 = 26–50%, 3 = 51–75%, and 4 = 76–100%).

p-value calculated using Mann-Whitney U test.

### Mononuclear Cell Isolation and Cryopreservation

Tonsils were cut into small pieces, ground through a 100μm cell strainer using a plunger of a plastic syringe and washed with PBS. Cell suspension was collected, spun down and resulting pellet resuspended in 35ml PBS. The obtained cell suspension was loaded on top of 15ml lymphoprep (Fisher Scientific) and mononuclear cells were isolated using ficoll gradient density centrifugation. Subsequently, mononuclear cells were frozen in FCS + 10% DMSO (Sigma Aldrich) freezing medium and stored in the gas phase of liquid nitrogen.

### Cell Preparation and Flow Cytometry

Frozen mononuclear cells were thawed and washed twice with IMDM + 10% FCS. Cells were stained with fixable viability dye (ThermoFisher) and antibodies targeting surface molecules at room temperature in FACS buffer (PBS + 2% FCS + 2mM EDTA) for 30 minutes whereafter cells were fixed for 10 minutes with 2% paraformaldehyde. Ki67 and T-bet were stained using eBioscience™ FoxP3/Transcription Factor Staining Buffer Set (Thermo Fisher Scientific) according to manufacturer’s instructions. A full list of antibodies used and staining panels can be found in **Supplementary Tables 1, 4, 9**. Stained samples were acquired on BD LSR Fortessa™ equipped with 355-, 405-, 488-, 561-, and 639-nm lasers.

### Flow Cytometry Analysis

FCS3.0 files were analyzed using FlowJo v. 10.6.1 (BD Biosciences). Automated compensation was calculated using single-stained compensation beads (BD Biosciences) on BD FACSDiva software and manually adjusted on FlowJo by looking at pairwise expression of all parameters. Automated cluster analysis and visualization of concatenated cell populations was performed using FlowJo plugins. Cell numbers of the populations of interest were reduced using DownSample (v. 3.3) function and samples belonging to the same patient group were concatenated using all compensated parameters. The cell numbers were adjusted to be equal between the patients within each group and comparable between the two patient groups (small *vs*. large tonsils) in the final concatenated file. The final cell number was 7 200 and 7 205 cells for ILCs and 18 000 and 18 005 cells for T cells and B cells for small and large tonsil group, respectively. UMAP (v. 2.1) was used for dimensionality reduction and PhenoGraph (v. 1.8) was used to investigate the population heterogeneity within each group and the results were visualized using ClusterExplorer (v. 1.1.6). Default settings were used for UMAP (Euclidean distance function, nearest neighbors = 15 and minimum distance = 0.5) and PhenoGraph (K = 30) algorithms. Parameters specific to the cell type of interest were selected to run UMAP and PhenoGraph and are specified in the **Supplementary Tables 1, 4, 9**.

### Statistics

Statistical analysis was performed using Prism software v. 6 (GraphPad). Statistical difference between data sets was assessed using Mann-Whitney *U* test. Two-tailed *P*-values < 0.05 were considered significant.

## Results

### Flow Cytometric Data Analysis Workflow

**Figure 1:**
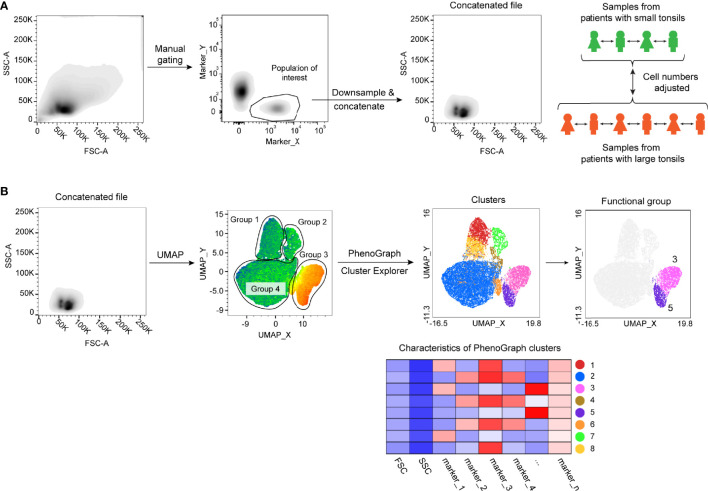
Flow cytometric data analysis workflow. **(A)** Schematics showing sample concatenation of cell populations derived from the same patient group. **(B)** UMAP and PhenoGraph analysis workflow of concatenated samples.

To address if immunological changes in tonsillar tissue correlate with the tonsil size in pediatric patients, we selected a cohort of 19 children with small tonsils and moderate OSA (n = 6) and with large tonsils and very severe OSA (n = 13) (**Table 1**), hereafter referred to as small and large tonsils. Three flow cytometric panels of surface and intracellular proteins were designed to identify the main ILC, T-cell and B-cell populations (**Supplementary Tables 1, 4, 9**). To reduce dimensionality and avoid bias related to manual gating, The FlowJo Plugins UMAP (25) and PhenoGraph (26) were applied to identify and project distinct lymphocyte clusters. First, major cell subsets of interest (Lin^-^CD127^+^ ILCs, CD3^+^ T cells, or CD3^-^CD19^+^CD20^+^ B cells) were manually gated on the basis of viable cells in all FCS files. Subsequently, viable cell populations from samples belonging to the same patient group were down-sampled and concatenated. Cell numbers were adjusted, so that individual patient samples were equally represented in each group and the final cell number in the concatenated files was comparable between both tonsil size groups according to the workflow presented in **Figure 1A**. UMAP and PhenoGraph algorithms were run using all compensated parameters specific to the cell type of interest (**Supplementary Tables 1, 4, 9**). We then analyzed groups of cells projected on the UMAP, followed by PhenoGraph analysis and the projection of the resulting clusters onto the UMAPs (**Figure 1B**; workflow). Based on the defining marker expression and pre-existing knowledge, clusters were then grouped into distinct functional groups. This allowed for a direct unbiased comparison of lymphocyte populations between the two tonsil size groups. The MFI values for each marker and cluster can be found in **Supplementary Data 1**.

### Heterogeneity of ILC Populations in Tonsils of Pediatric Patients With OSA

We first set out to investigate the heterogeneity of ILC populations in the tonsils. For this, we designed a panel of surface markers to identify the main ILC lineages, their maturation/activation status as well as tissue residency phenotype (**Supplementary Table 1**). ILCs were readily detectable across all samples as Lin^-^CD3^-^CD127^+^ lymphocytes (**Supplementary Figure 1A**) as previously reported (19).

**Figure 2:**
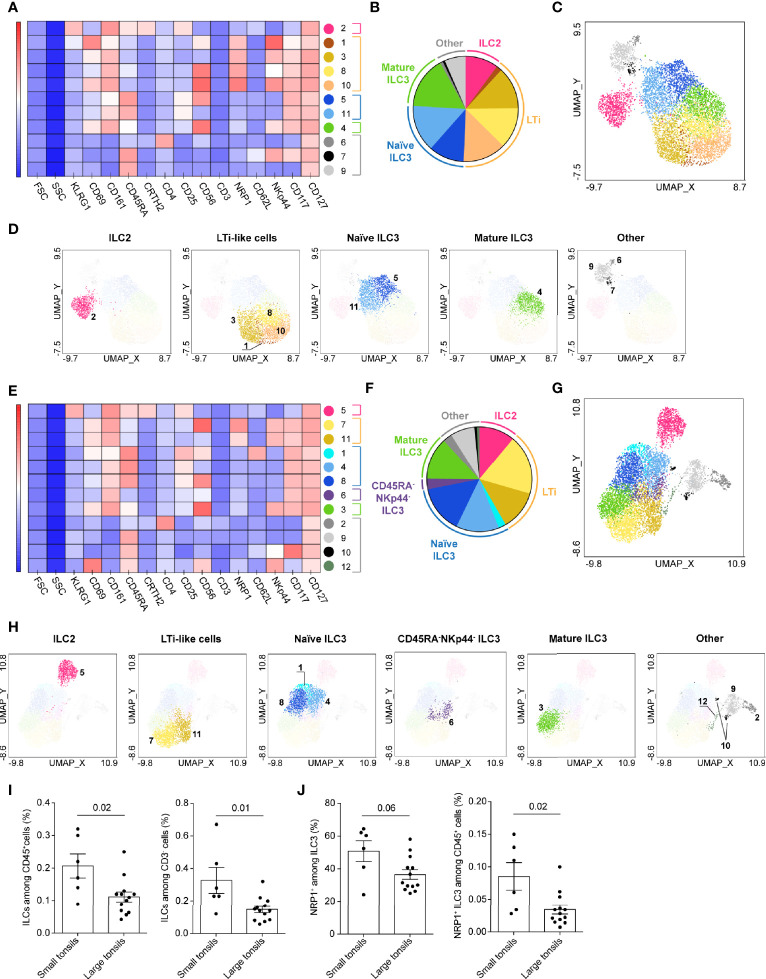
CD127^+^ ILC clusters in tonsils of pediatric patients with OSA. **(A–H)** CD127^+^ ILC clusters in small **(A–D)** and large **(E–H)** tonsils from patients with OSA, identified by PhenoGraph in the concatenated files. **(A, E)** Heatmap of marker expression intensity scaled individually for each marker. **(B, F)** Diagram showing proportions of PhenoGraph clusters and combined functional groups. **(C, D, G, H)** PhenoGraph clusters overlaid on the corresponding UMAP together **(C, G)** or separated by functional group **(D, H)**. **(I)** Frequency of ILCs among total living CD45^+^ cells and CD3^-^ cells. **(J)** Frequency of NRP1^+^ ILC3 subset among ILC3 and total living CD45^+^ cells. **(I, J)** Bars and error bars indicate mean ± SEM. Statistical significance was calculated using Mann-Whitney *U* test.

For both small and large tonsils, UMAP projected the CD127^+^ ILCs in three major groups based on their lineage characteristics: CRTH2^+^ ILC2, CD117^+^ ILC3 including LTi-like cells, and CD161^-^ ILCs (**Supplementary Figures 1B, C**). Using PhenoGraph analysis, 11 clusters of CD127^+^ ILCs were detected in the small tonsils (**Figures 2A–D**; **Supplementary Table 2**), and 12 in the large tonsils (**Figures 2E–H** and **Supplementary Table 3**). In both disease groups, CRTH2^+^ ILC2 were allocated to one cluster, hence making up a highly homogenous cell population, while CD117^+^ ILC and CD161^-^ ILC groups displayed additional heterogeneity in terms of the markers analyzed (**Figures 2C, G**). In line with previous observations (28, 29), tonsillar ILC2 showed largely uniform expression of CRTH2, CD161, CD25 and KLRG1 (**Figures 2A, E** and **Supplementary Tables 2, 3**). Of note, most ILC2 in both small and large tonsils did not express CD69, suggesting that these are circulating and/or less activated cells (**Figures 2A, E** and **Supplementary Tables 2, 3**).

Using single-cell RNA sequencing (scRNA-seq), Björklund and Forkel et al. have previously described transcriptionally distinct sub-populations of ILC3 in human tonsils, defined by their expression of CD62L and NKp44 (19). CD62L^+^ cells co-expressed CD45RA and delineated a naïve-like subset of ILC3, while NKp44 expression characterized mature, IL-22 producing ILC3. Indeed, the marker distribution pattern of the CD117^+^ ILC group (**Supplementary Figures 1B, C**) demonstrated mutually exclusive expression of CD45RA and NKp44 in both disease groups. PhenoGraph distinguished seven clusters of CD117^+^ ILCs in small and large tonsils alike. Based on existing knowledge (19, 22, 24), the CD117^+^ ILC clusters were combined into three major functional groups: NRP1^+^ LTi-like cells, CD45RA^+^ naïve ILC3-like and NKp44^+^ mature ILC3 (**Figures 2A–H** and **Supplementary Tables 2, 3**). Additionally, a separate cluster of CD45RA^-^NKp44^-^ ILC3 was identified in the large-tonsil group, likely representing a transient stage between naïve and mature ILC3 (**Figures 2F, H** and **Supplementary Table 3**). While naïve CD45RA^+^ and transient CD45RA^-^NKp44^-^ ILC3 clusters contained equal proportions of CD69^-^ and CD69^+^ cells, mature NKp44^+^ ILC3 displayed predominantly a tissue-resident or activated CD69^+^ phenotype (**Supplementary Tables 2, 3**).

PhenoGraph analysis detected several clusters of LTi-like cells (**Figures 2A–H** and **Supplementary Tables 2, 3**). The detected heterogeneity was attributed to variable expression of CD56 and NKp44 on the individual LTi-like-cell populations (**Figures 2A, E** and **Supplementary Tables 2, 3**). Although some CD69^-^ cells were present in the LTi-like cell clusters (**Supplementary Tables 2, 3**), most of the LTi-like cells expressed CD69, highlighting their tissue-resident or activated phenotype (**Figures 2A, E** and **Supplementary Tables 2, 3**).

Within the CD161^-^ ILC group, PhenoGraph identified three separate cell clusters in the small tonsils and four in large tonsils (**Figures 2A–H** and **Supplementary Tables 2, 3**). Besides the CD161^-^CD117^+^ ILC3-like cells and CD161^-^CRTH2^-^CD117^-^ ILC1-like, CD4-expressing CRTH2^-^CD117^-^ ILC1-like cells were identified in both tonsil groups, although this cluster was very small in small tonsils (**Figures 2A–H** and **Supplementary Tables 2, 3**). Roan et al. have previously described a distinct population of CD4^+^ ILC1 that was increased in the peripheral circulation of the individuals with systemic sclerosis (30). That population was characterized by the absence of surface TCR and CD3ϵ expression, but showed intracellular expression of CD3ϵ. Tonsillar CD4^+^ CRTH2^-^CD117^-^ ILC1-like cells were largely CD3^-^, but contained a subset of CD3^lo^ cells. This indicates that the CD161^-^CD4^+^ ILC cluster might contain a mixture of CD4^+^ ILC1 and highly activated CD4^+^ T cells that have downregulated surface TCR and CD3.

Finally we evaluated the differences in ILC composition between small and large tonsils. Significantly lower percentage of CD127^+^ ILCs out of CD45^+^ cells, as well as among CD3^-^ lymphocytes, was detected in large as compared to small tonsils (**Figure 2I**). While we observed similar frequencies of ILC2 and ILC1-like cells, a lower frequency of LTi-like cells was found in the concatenated data set from large tonsils (**Supplementary Tables 2, 3**). The frequency of NRP1^+^ ILC3 out of CD45^+^ cells was indeed significantly lower and tended to be lower as a proportion of total ILC3, in large *versus* small tonsils (**Figure 2J**).

In summary, we here provide an overview of ILC heterogeneity in human tonsils and identify skewed ILC3 composition in enlarged tonsils.

### Heterogeneity of T Cells in Tonsils of Pediatric Patients With OSA

**Figure 3:**
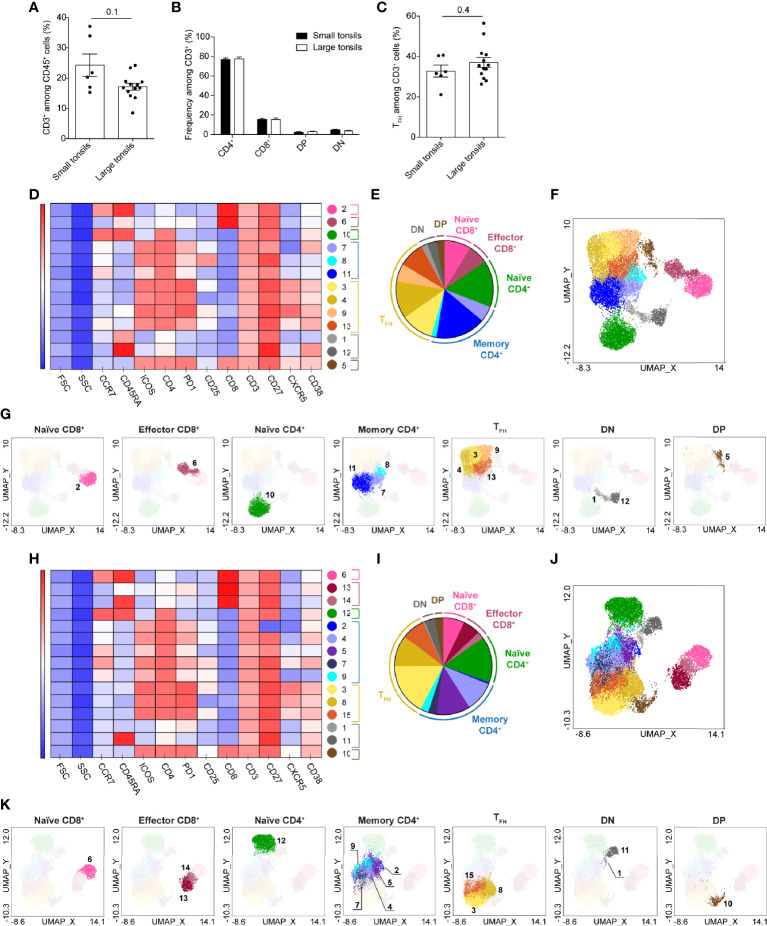
T-cell clusters in tonsils of pediatric patients with OSA. **(A)** Frequency of CD3^+^ T cells among total living CD45^+^ cells. **(B)** Frequency of CD4^+^, CD8^+^, DP and DN T cells among total CD3^+^ cells. **(C)** Frequency of T_FH_ cells among total T cells. **(A–C)** Bars and error bars indicate mean ± SEM. Statistical significance was calculated using Mann-Whitney *U* test. **(D–K)** T-cell clusters in small **(D–G)** and large **(H–K)** tonsils from patients with OSA, identified by PhenoGraph in the concatenated files. **(D, H)** Heatmap of marker expression intensity scaled individually for each marker. **(E, I)** Diagram showing proportions of PhenoGraph clusters and combined functional groups. **(F, G, J, K)** PhenoGraph clusters overlaid on the corresponding UMAP together **(F, J)** or separated by functional group **(G, K)**.

Next, we sought out to examine the composition of adaptive lymphocytes in the tonsillar tissues. A panel of surface markers was selected to dissect B- and T-cell populations and address their activation, maturation and functional status (**Supplementary Table 4**). T cells were manually gated as live CD45^+^CD3^+^ lymphocytes (**Supplementary Figure 2A**). As small and large tonsils showed a comparable composition of CD3^+^ T cells and the main T cell lineages including T_FH_ cells (**Figures 3A–C** and **Supplementary Figure 2A**) we proceeded to assess the heterogeneity of T cells in the two tonsil groups (**Figures 3D–K**).

UMAP projected the T cells in five main groups in both small and large tonsils: CD45RA^+^ and CD45RA^-^ CD4^+^, CD4^+^CD8^+^ double positive (DP), CD4^-^CD8^-^ double negative (DN) and CD8^+^ T cells (**Supplementary Figure 2**). Interestingly, DP and DN T cells were located closer to CD4^+^ T cells than to CD8^+^ T cells in both patient groups, with DP T cells projected close to CD45RA^-^ and DN to CD45RA^+^ CD4^+^ T cells. CD8^+^ T cells were projected furthest from other T-cell subsets, with CD45RA^-^ and CD45RA^+^ CD8^+^ cells located more closely to each other as compared to CD45RA^-^ and CD45RA^+^ CD4^+^ cells. This may be attributed to the inclusion of T_FH_-centric surface markers during the flow-cytometric panel design, as this would allow a more in-depth analysis of CD4^+^ T-cell populations.

PhenoGraph algorithm identified 13 and 15 clusters of T cells in small and large tonsils, respectively (**Figures 3D–K** and **Supplementary Tables 5, 6**). Based on the surface marker expression (**Figures 3D, H** and **Supplementary Tables 5, 6**), the clusters identified were categorized into 7 major functional groups: naïve, memory and T_FH_ CD4^+^ cells, naïve and effector CD8^+^ cells, as well as DN and DP T cells (**Figures 3E–G, I–K**). Identified clusters were superimposed onto the UMAP described above (**Figures 3F, G, J, K**). In both small and large tonsils, the CD45RA^+^CD4^+^ T-cell group consisted of a homogenous naïve cell cluster that was defined by the expression of CD45RA and CCR7 (**Figures 3D, F, G, H, J, K** and **Supplementary Tables 5, 6**).

The CD45RA^-^CD4^+^ T-cell groups contained 7 and 8 cell clusters in small and large tonsils, respectively, representing functional groups of T_FH_ cells and memory helper T cells. The T_FH_-cell clusters showed variable expression of the defining markers ICOS, PD-1 and CXCR5 and were largely CCR7^-^. Moreover, the distinct T_FH_ clusters differed in their expression of IL-2 receptor α chain, CD25 (**Figures 3D, H** and **Supplementary Tables 5, 6**). Interestingly, DP T cells displayed a phenotype highly similar to T_FH_ cells, characterized by CXCR5, PD1 and ICOS expression, but without increased size, thus unlikely representing cell doublets (**Figures 3D, H** and **Supplementary Tables 5, 6**). Three and five non-T_FH_ memory helper T-cell clusters were identified in the small and large tonsils, respectively. This difference was not due to an enrichment of memory CD4^+^ T cells in large tonsils, as no differences in the frequencies of CD45RA^-^CD4^+^ T cells or T_FH_ cells were detected between the groups at the patient level (**Figure 3C** and **Supplementary Figure 2E**).

DN T cells contained two clusters in the small and large tonsils (**Figures 3D–K**). Both clusters expressed CD27, were CCR7^-^ and did not express any T_FH_-related surface markers. These clusters differed from each other in their expression of CD38 and CD45RA (**Figures 3D–K**), as well as PD1 in the large tonsils (**Figures 3H–K**).

Within the CD8^+^ T-cell group, two clusters were identified in small tonsils and three in large tonsils (**Figures 3D–K**). While naïve CD8^+^ T cells represented a separate cluster in both groups, PhenoGraph identified CD45RA^-^ memory and CD45RA^+^CCR7^-^ TEMRA cells as one cluster in patients with small tonsils (**Figures 3D–K** and **Supplementary Tables 5, 6**). These differences were not reflected in frequency changes of the described cell subsets between the two patient groups (**Supplementary Figure 2F**) and could simply be attributed to the uniformity of surface marker expression therein.

Taken together, automated clustering projection and analysis using UMAP and PhenoGraph, respectively, allowed unbiased identification of T-cell diversity in small and large tonsils from pediatric patients with OSA, which could not be readily identified using manual gating.

### Heterogeneity of B Cells in Tonsils of Pediatric Patients With OSA

**Figure 4:**
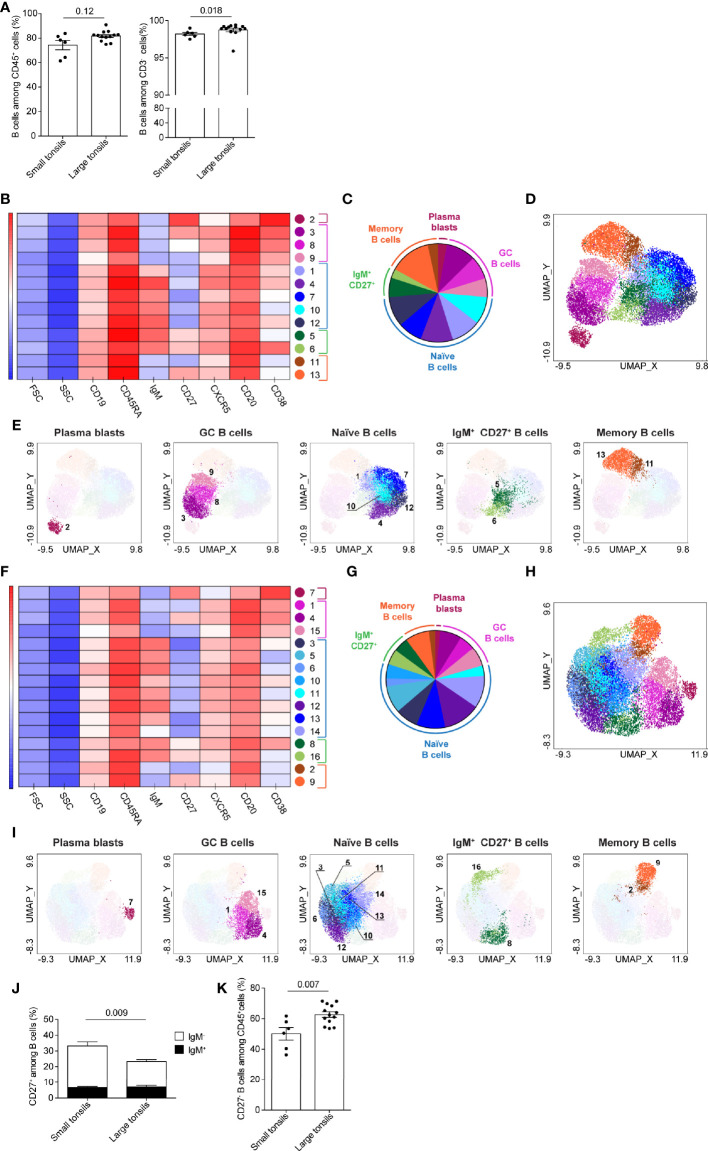
B-cell clusters in tonsils of pediatric patients with OSA. **(A)** Frequency of B cells among total living CD45^+^ cells and CD3^-^ cells. **(B–I)** B-cell clusters in small **(B–E)** and large **(F–I)** tonsils from patients with OSA, identified by PhenoGraph in the concatenated files. **(B, F)** Heatmap of marker expression intensity scaled individually for each marker. **(C, G)** Diagram showing proportions of PhenoGraph clusters and combined functional groups. **(D, E, H, I)** PhenoGraph clusters overlaid on the corresponding UMAP together **(D, H)** or separated by functional group **(E, I)**. **(J)** Frequency of CD27^+^ cells among total B cells; black bars indicate IgM^+^ and white bars indicate IgM^-^ subsets. **(K)** Frequency of CD27^-^ B cells among total living CD45^+^ cells **(A, J, K)** Bars and error bars indicate mean ± SEM. Statistical significance was calculated using Mann-Whitney *U* test.

To assess the composition of tonsillar B cells in pediatric patients with OSA, B cells were manually gated based on their CD19 and CD20 expression (**Supplementary Figures 2A, 3A**). As previously reported (31), no CD20^-^CD38^hi^ plasma cells were detected in tonsillar tissue, as all CD38^hi^CD3^-^ cells displayed low CD20 expression (**Supplementary Figure 3B**). Patients with large tonsils demonstrated a tendency for higher frequency of total B cells than patients with small tonsils (**Figure 4A**). This difference was statistically significant, when only the CD3^-^ population was accounted for.

B-cells were projected in four main groups in UMAPs generated from small tonsils: CD38^hi^CD20^lo^ cells (likely representing plasmablasts), CD38^+^CD20^hi^ germinal center (GC), CD27^+^IgM^-^ memory and IgM^+^ B cells (**Supplementary Figure 3C**). In large tonsils an additional cell group of CD27^+^IgM^+^ cells was detectable, that localized between the IgM^+^ and CD27^+^IgM^-^ cell clusters (**Supplementary Figure 3D**).

13 and 16 clusters of B cells were identified by PhenoGraph in small and large tonsils, respectively (**Figures 4B–I**). Based on the surface marker expression (**Figures 4B, F** and **Supplementary Tables 7, 8**), B-cell clusters were categorized into five functional groups: plasmablasts, GC B cells, naïve and memory B cells, as well as IgM^+^CD27^+^ B cells (**Figures 4C, E, G, I**). In both, small and large tonsils, plasmablasts were represented by one homogenous cluster of cells, defined by their CD20^lo^CD27^hi^CD38^hi^ phenotype (**Figures 4B–I** and **Supplementary Tables 7, 8**). Additionally, plasmablasts did not express IgM and were largely CXCR5^-^. The GC B-cell functional group contained three clusters that showed variance in the expression intensity of CD38, CD27, CXCR5, CD19 and/or CD20 in small as well as large tonsils (**Figures 4B–I** and **Supplementary Tables 7, 8**). This phenotypic variability might reflect different maturation stages of GC B cells in the process of plasma-cell, or memory B-cell development. Specifically, the expression intensity of the chemokine receptor CXCR5 might be indicative of the migration status of the given cluster through B-cell follicles (32).

IgM^-^ memory B cells and IgM^+^CD27^+^ B cells, each represented by two clusters, showed high similarity between small and large tonsils (**Figures 4B–I** and **Supplementary Tables 7, 8**). While we lack additional markers to fully dissect the IgM^+^CD27^+^ B cell clusters, they showed differential expression of CD38 suggesting that one of these clusters might be IgM^+^ GC B cells or marginal zone B cells (33) while the other may represent IgM^+^ memory B cells (34). In both small and large tonsils, the CD27^-^IgM^-^ memory B-cell clusters displayed lower expression of CXCR5 than their CD27^+^ counterparts (**Figures 4B, F** and **Supplementary Tables 7, 8**). When we compared the frequency of CD27^+^ B cells in small and large tonsils on the individual patient level, we detected a significantly higher percentage of these cells in small tonsils (**Figure 4J**; **Supplementary Figure 3E**). The difference observed could be contributed to conventional memory B cells, as frequencies of IgM^+^CD27^+^ memory B cells were the same in both patient groups (**Figure 4J**).

PhenoGraph identified five naïve B-cell clusters in small tonsils *vs*. 8 clusters in large tonsils (**Figures 4B–I** and **Supplementary Tables 7, 8**). In terms of population size, naïve B-cells were more prominently represented in the large tonsils than in the small ones (**Figures 4C, G**). Moreover, through manual gating we identified an enrichment of CD27^-^ B cells among total CD45^+^ cells in pediatric patients with large tonsils (**Figure 4K**). However, the frequencies of IgM^+^ cells within the CD27^-^ B-cell populations were the same between the two patient groups (**Supplementary Figure 3F**).

In summary, we identified enrichment of IgM^+^CD27^-^ naïve B-cell populations in large tonsils from pediatric patients with very severe OSA. This is consistent with the observed increase in frequency of total B cells and CD27^-^ B cells in this patient group.

### Atypical Memory B-Cell Populations in Pediatric Patients With OSA

A unique subset of CD27^-^ B cells harboring memory potential was described in chronic inflammation and suggested to be driven by infections as well as autoimmunity (35–38). These tissue-like memory or atypical memory B cells were characterized by the expression of Fc receptor-like (FcRL) proteins FcRL4 and/or FcRL5, CD11c and T-bet (35, 38–40). To investigate the atypical B cell populations in relation to our finding on increased CD27^-^ B cells in large tonsils, we developed a flow-cytometric panel including the defining markers as well as Ki67 (**Supplementary Table 9**). Concatenated group-specific samples were generated from total B-cell populations (**Supplementary Figure 4A**). Based on the markers analyzed, two main B-cell groups were observed in UMAPs generated from large and small tonsils: CD38^hi^CD20^lo^ plasmablasts and other B cells that were projected in close proximity to each other (**Supplementary Figures 4B, C**).

**Figure 5:**
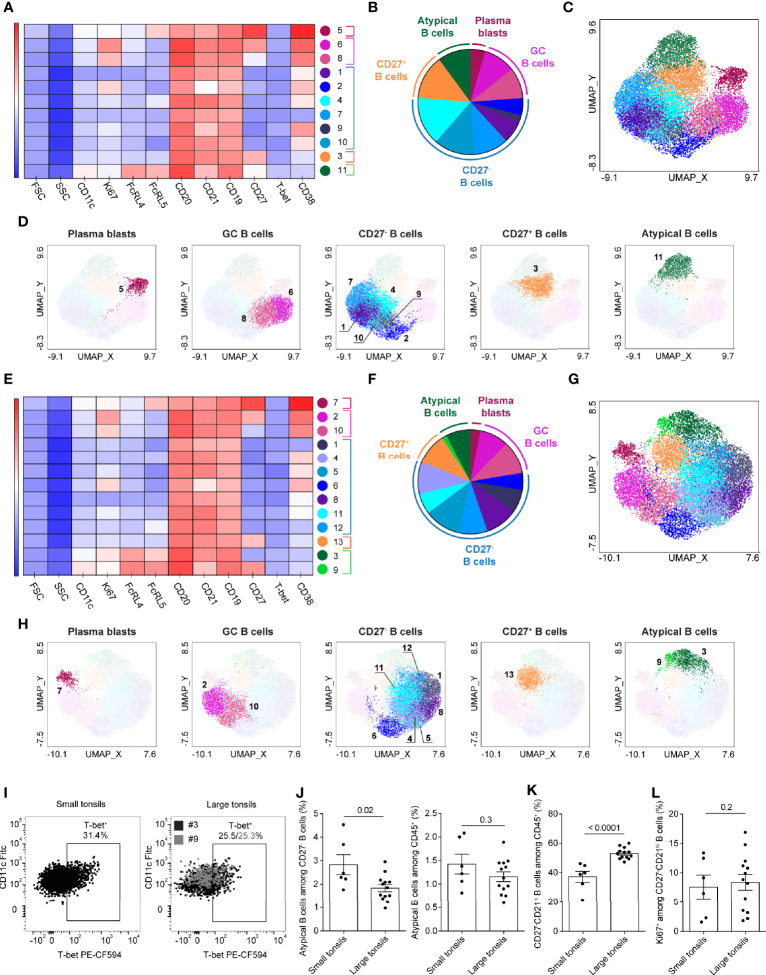
Atypical memory B cells in tonsils of pediatric patients with OSA. **(A–H)** B-cell clusters in small **(A–D)** and large **(E–H)** tonsils from patients with OSA, identified by PhenoGraph in the concatenated files. A different panel of markers to those in **Figure 4** was applied to allow a better resolution of atypical B-cell populations. **(A, E)** Heatmap of marker expression intensity scaled individually for each marker. **(B, F)** Diagram showing proportions of PhenoGraph clusters and combined functional groups. **(C, D, G, H)** PhenoGraph clusters overlaid on the corresponding UMAP together **(C, G)** or separated by functional group **(D, H)**. **(I)** T-bet and CD11c expression in the atypical memory B-cell clusters identified by PhenoGraph in small and large tonsils. **(J)** Frequency of atypical memory B cells among CD19^+^CD20^+^ B cells and total living CD45^+^ cells. **(K)** Frequency of CD27^-^CD21^hi^ B cells among total living CD45^+^ cells. **(L)** Frequency of Ki67^+^ cells among CD27^-^CD21^hi^ B cells. **(I–L)** Bars and error bars indicate mean ± SEM. Statistical significance was calculated using Mann-Whitney *U* test.

PhenoGraph detected 11 and 13 clusters in small and large tonsils, respectively (**Figures 5A–H**). These clusters could be attributed to five major functional groups: plasmablasts, GC B cells, CD27^-^ B cells, CD27^+^ B cells and atypical memory B cells (**Figures 5B, D, F, H**). The atypical memory B cells were characterized by co-expression of FcRL4, FcRL5 and CD11c, and were largely CD27^-^ (**Figures 5A, E** and **Supplementary Tables 10, 11**). In small tonsils, atypical memory B cells comprised a single population that was heterogeneous in terms of T-bet expression, with 30% of cells being positive for the transcription factor (**Figures 5A–D**, **I** and **Supplementary Table 10**). In large tonsils, we found both CD27^-^ and CD27^+^ atypical B-cell clusters (#3 and 9, respectively), where the CD27^+^ cluster also had increased expression of CD11c (**Figures 5E-H** and **Supplementary Table 11**) and might represent a transient step in the development of atypical memory B cells. Similar to their counterparts in small tonsils, atypical memory B cells in large tonsils were diverse in terms of T-bet expression, with 25% of cells being T-bet^+^ (**Figure 5I**). While in small tonsils higher T-bet expression intensity appeared to correlate with increased surface CD11c expression on atypical memory B cells, this correlation was not as prominent in large tonsils (**Figure 5I**).

To compare the frequency of atypical memory B cells between both disease groups on an individual patient level we manually gated CD27^-^CD21^-^FCRL4^+^FCRL5^+^CD11c^lo^ cells (**Supplementary Figure 4A**). Although atypical memory B cells were significantly increased among CD27^-^ B cells in patients with small tonsils, the increase was not observed among total CD45^+^ cells (**Figure 5J**).

Based on the CD27 and CD21 expression, four different B cell subsets have been previously described: CD27^+^CD21^hi^ resting memory, CD27^+^CD21^lo^ activated memory, CD27^-^CD21^lo^ tissue-like memory and CD27^-^CD21^+^ naïve B cells (41, 42). In line with our observations described above (**Figure 4K** and **Supplementary Figure 3E**), we observed a strong enrichment of CD27^-^CD21^hi^ naïve B-cell population in patients with large tonsils (**Figure 5K**). This was not associated with increased Ki67 expression (**Figure 5L**) thus, is unlikely to be a consequence of increased proliferation of naïve B cells in patients with large tonsils and very severe OSA.

## Discussion

In the present study we addressed the heterogeneity of lymphocyte populations, including ILCs, T cells and B cells in tonsils of pediatric patients categorized into two groups: small tonsils with moderate OSA, and large tonsils with very severe OSA. Multi-color flow cytometry followed by unbiased high-dimensional single-cell mapping was used to delineate cell subsets and compare them between small and large tonsils. Unbiased analysis was chosen to ensure the detection of cell populations and transient developmental stages in cell development that risk being overlooked when manually gated.

A major difference in lymphocyte composition between small and large tonsils was the shift in ILC and B-cell composition. Large tonsils showed a significant increase in CD27^-^ B cells and a relative reduction of ILCs. Studies have also shown an increase of naïve B-cells in tonsils from healthy EBV-carriers (43). The hypothesis of a viral infection causing tonsillar hyperplasia has been addressed but not firmly proven. One study found acute infection with human adenovirus as a possible contribution to tonsillar hyperplasia among young patients (44). As CD27^-^ B cells encompass both naïve and atypical memory B cells, the latter appearing in several chronic infectious diseases including malaria (45), tuberculosis (46) and HIV (41), we addressed the presence of atypical memory B cells but found similar frequencies of these cells in small and large tonsils. Upon closer inspection, naïve CD27^-^CD21^hi^ B cells constituted the population driving the increase in total B cells in large tonsils. This enrichment was not paralleled by increased proliferation, as demonstrated by Ki67 expression. Hence, we hypothesize that the increase in naïve B cells observed in large tonsils in severe OSA might be a consequence of an impaired B-cell differentiation process. This should be addressed in more mechanistic studies.

This raised a question about potential defects in cells supporting B cell differentiation. In this perspective, the reduction of ILC3 with a phenotype of LTi cells in large OSA tonsils is interesting given the role for LTi cells in the establishment of lymphoid structures during fetal development in mice (22). However, the role for LTi cells in already established secondary lymphoid structures after birth and in humans is less well characterized. ILC3 have also been shown to support B cell differentiation in the spleen *via* the production of the B cell activation factor (BAFF), the ligand for the co-stimulatory receptor CD40 (CD40L) and the Notch ligand delta like 1 (DLL1) (47). The relative reduction of such B-cell supporting ILC3 could contribute to reduced signals for B cell differentiation and accumulation of naïve B cells as we observed herein. This finding remains to be further explored.

We additionally explored potential sources of T cell help to B cells but found no changes in the T cell compartment, including T_FH_ cells, in large *versus* small tonsils. Matsumiya et al. have previously demonstrated that, in adults, tonsils from patients with OSA contain considerably larger GC areas with increased frequency of GC B cells compared to those from patients with recurrent tonsillitis (RT) (48). Although OSA tonsils contained higher numbers of T_FH_ cells than RT tonsils, OSA tonsil-derived T_FH_ cells were inferior in terms of providing B cell help. Functional impairment of this nature was linked to the reduced expression of the transcription co-activator Bob1 and decreased IL-4 production in T_FH_ cells from OSA tonsils (48). Dan et al. published similar results in a study on tonsils from children with RT compared to OSA tonsils, where RT-tonsils showed smaller GC and a lower frequency of GC-T_FH_ cells (49). They also found a lower frequency of GC B cells in RT tonsils compared to OSA tonsils, but higher frequencies of naïve B cells in RT tonsils (49).

In addition to the accumulation of naïve B cell in large tonsils, we observed heterogeneity of naïve B cells. The naïve B cell heterogeneity was mainly driven by variable expression of CD38 and CXCR5, possibly revealing different stages transiting to GC B cells. Indeed a recent scRNA-seq study of human tonsil lymphocytes revealed a subset of pre-GC cells that was transcriptionally similar to naïve B cells (50). Similarly, we observed GC B cell heterogeneity. While we are careful in interpreting GC B cell data in our cohort, due to the observed loss of viability after freeze-thawing, we did detect variable CD27 and CXCR5 expression. This finding, which should be followed up in freshly isolated tonsils, could be indicative of transitional states in their differentiation into plasma and/or memory B cells. Again this is supported by recent scRNAseq of human tonsils, which identified a pre-GC, four GC and a pre-plasmablast subset with variable expression of *CD27* (50). Another GC-focused study of human tonsils also revealed significant heterogeneity with the identification of 13 transcriptionally distinct GC B cell subsets (51). Although we cannot compare our more limited analysis of surface proteins to scRNAseq analysis, the latter can inform about additional relevant B cell proteins to be explored in in tonsil hyperplasia and OSA. While memory B cells express CD27, other markers of B cell differentiation deserve further investigation. CCR6 has been suggested as a marker of memory B cell precursors in germinal centers of both mice and in human tonsils (52). Furthermore, exploration of transcriptional programs regulated by paired box protein 5 (Pax5) and B lymphocyte induced maturation protein 1 (BLIMP-1), governing early B cell differentiation and plasma cell formation, respectively (53), is warranted. Such future studies could reveal the mechanisms underlying the tonsil hyperplasia, seemingly driven by accumulation of naive B cells, possibly as a consequence of defective B cell differentiation into memory or plasma B cells. This is particularly interesting given a possible association between removal of tonsils in childhood and immune dysregulation later in life (54).

The variable expression of CXCR5 on naïve and GC B cells is interesting given its role in GC migration in response to the high levels of CXCL13 produced by follicular dendritic cells, as predominantly demonstrated in mice (32). Indeed, in human lymph nodes, B cells show varying CXCR5 expression across areas with different CXCL13 expression, suggesting that CXCR5 is dynamically regulated by CXCL13 levels in a spatial manner (55). As B cell migration through the follicle is critical for differentiation, studies of other critical chemotactic receptors and their ligands in OSA is warranted. This includes CCR7 and CXCR4, important for naïve B cell entry to lymph nodes (32, 55), and g-protein coupled receptor (GPR)-183 (Epstein-Bar virus induced gene 2; EBI2), regulating B cell migration to inter- and outer follicular areas in mice (32). The accumulation of naïve B cells in the large tonsils could indicate defects in lymph node exit mechanisms. Critical in this process is the sphingosine-1-phosphate receptor 1 (S1PR1) which responds to an increasing gradient of S1P across the follicular boundary into the cortical sinus (55). Given that the S1PR-agonist Fingolimod is successfully used to inhibit lymphocyte trafficking in multiple sclerosis (56), studies of S1P subtypes, its receptors and other chemotactic molecules in OSA could open up for new therapy targets in this disease.

Finally, our study comes with some limitations and strengths. Our finding of increased B cell frequencies in large tonsils and very severe OSA was observed using two distinct flow cytometry panels in two independent sets of experiments, each validating the other. However, while our cohort was clinically well characterized, including polysomnography, we compared significantly enlarged tonsils from children with very severe OSA to small tonsils from children with moderate OSA. This comparison is valuable and unique since it unusual to perform surgery on children with small tonsils. However, we do not have a healthy control group since it is unethical to do surgery on children who do not suffer from OSA or tonsillar hyperplasia. We also found it unsuitable to use tonsils surgically removed because of RT as bacterial infections and biofilm are not only associated with immune activation but also tonsillar hyperplasia (57, 58). Nevertheless, a lack of a complete healthy control group is a limitation of this study. As for GC B cells, these cells are prone to apoptosis as a physiological part of their selection process (59) and we did indeed detect a consistent reduction of GC B cells after freezing/thawing of our tonsil samples (data not shown). We also observed a consistent reduction of T cells after freeze/thawing (data not shown). Since the markers expressed by these subsets were not affected by freezing/thawing, they can still be evaluated for studies of heterogeneity. However, validation of the current findings, and further elaborated investigations of B cells in a larger cohort of fresh tonsil samples is needed to fully understand the implications of our findings. Although our data cannot provide mechanistic insight into disease onset or progression, they nevertheless present an overview of lymphocyte subset disturbances in OSA. Therefore, our findings may aid hypothesis generation with the aim of spurring additional research in this important field.

In summary, we here performed a multidimensional investigation of ILCs, T cells and B cells in OSA tonsils, unveiling perturbed ILC3 and B cell frequencies in large *versus* small tonsils. Our study provides an overview of human tonsil lymphocytes but also reveals potential new targets for further exploration of tonsil hyperplasia in OSA.

## Data Availability Statement

The original contributions presented in the study are publicly available. This data can be found here: FlowRepository.org, accession number FR-FCM-Z4LF.

## Ethics Statement

The studies involving human participants were reviewed and approved by Swedish Ethical Review Authority. Written informed consent to participate in this study was provided by the participants’ legal guardian/next of kin.

## Author Contributions

AC formulated research questions, performed, analyzed and interpreted experiments and contributed to writing the manuscript. IS selected the patient cohort, formulated research questions, performed experiments, interpreted clinical data and contributed to writing the manuscript. AVA contributed to experimental design, performed and analyzed experiments. AD contributed to experimental design and data interpretation. JF selected the patient cohort and interpreted clinical data. MF contributed to experimental design and data interpretation. DF selected the patient cohort, formulated research questions, interpreted clinical data and contributed to writing the manuscript. JM formulated research questions, contributed to experimental design, interpreted the data and wrote the manuscript. AR formulated research questions, designed and executed the experiments, analyzed and interpreted the data and wrote the manuscript. All authors contributed to the article and approved the submitted version.

## Funding

AC, AVA, JM, and AR were supported by funding from Karolinska Institutet, the Swedish Research Council, the Swedish Cancer Society, the Swedish Foundation for Strategic Research and the Knut and Alice Wallenberg Foundation. AR was additionally funded by the German Research Foundation (Deutsche Forschungsgemeinschaft) postdoctoral fellowship (RA 2986/1-1). DF and IS were supported by funding from Uppsala County Council (ALF-agreement).

## Conflict of Interest

The authors declare that the research was conducted in the absence of any commercial or financial relationships that could be construed as a potential conflict of interest.

## Publisher’s Note

All claims expressed in this article are solely those of the authors and do not necessarily represent those of their affiliated organizations, or those of the publisher, the editors and the reviewers. Any product that may be evaluated in this article, or claim that may be made by its manufacturer, is not guaranteed or endorsed by the publisher.
