# Supplementary material for: The Tonsil Lymphocyte Landscape in Pediatric Tonsil Hyperplasia and Obstructive Sleep Apnea
Source: Front Immunol. 2021 Oct 22;12:674080. doi: 10.3389/fimmu.2021.674080 (PMC8570126; doi:10.3389/fimmu.2021.674080)
Supplement: Supplementary file 1 [file DataSheet_1.docx]

Supplementary Material

# Supplementary Tables

| **Antigen** | **Clone** | **Fluorophore** | **Manufacturer** |
| --- | --- | --- | --- |
| Dead Cell Marker | - | Green | ThermoFisher |
| CD1a | HI149 | FITC | Biolegend |
| CD14 | TÜK4 | FITC | Dako |
| CD19 | 4G7 | FITC | BD |
| CD34 | 581 | FITC | Biolegend |
| CD94 | DX22 | FITC | Biolegend |
| CD123 | 6H6 | FITC | Biolegend |
| BDCA2 | AC144 | FITC | Miltenyi Biotec |
| FcεRIα | AER-37 | FITC | Biolegend |
| TCRαβ | IP26 | FITC | Biolegend |
| TCRγδ | B1 | FITC | Biolegend |
| KLRG1 | 13F12F2 | APC | eBioscience/ ThermoFisher |
| CD69 | FN50 | A700 | Biolegend |
| CD161 | 191B8 | APC-A750 | BeckmanCoulter |
| CRTH2 | BM16 | v450 | BD |
| CD4 | RPA-T4 | BV510 | Biolegend |
| PD1 | EH12.1 | BV605 | BD |
| CD25 | BC96 | BV650 | Biolegend |
| CD56 | HCD56 | BV711 | Biolegend |
| CD3 | OKT3 | BV785 | Biolegend |
| NRP1 | 12C2 | PE | Biolegend |
| CD62L | DREG56 | ECD | BeckmanCoulter |
| NKp44 | Z231 | PE-Cy5 | BeckmanCoulter |
| CD117 | 104D2D1 | PE-Cy5.5 | BeckmanCoulter |
| CD127 | R34.34 | PE-Cy7 | BeckmanCoulter |
| CD45 | HI30 | BUV395 | BD |
| CD45RA | HI100 | BUV737 | BD |

**Supplementary Table 1.** Staining panel used to identify ILC sub-populations in small and large tonsils. Markers used to run UMAP and PhenoGraph algorithms on Lin^-^CD127^+^ ILCs are underlined.

| **No.** | **Cluster** | **Phenotype** | **Tissue resident/activated (CD69^+^)** | **Frequency (%)** |
| --- | --- | --- | --- | --- |
| *1* | *CD56^lo^ LTi-like cells* | *NRP1^+/-^CD161^+^NKp44^++^CD117^+^CRTH2^-^CD45RA^-^ & CD56^lo^* | *Resident/activated* | *1.69* |
| *2* | *ILC2* | *KLRG1^+^CD161^+^CD45RA^+/-^CRTH2^+^CD117^-^CD25^+^* | *CD69^-^ 80%* | *10.1* |
| *3* | *CD56^-^ LTi-like cells* | *NRP1^+^CD161^+^NKp44^+^CD117^+^CRTH2^-^CD45RA^-^ & CD56^-^* | *Resident/activated 80%* | *13* |
| *4* | *CD56^+^ mature ILC3* | *NKp44^+^NRP1^-^CD161^+^CD45RA^+/-^CD62L^-^CD117^+^ & CD56^+^* | *Resident/activated 87%* | *16* |
| *5* | *CD56^+^ naïve ILC3* | *CD45RA^+^CD25^+^CD161^+/-^CD62L^-/+^NKp44^-^CD117^+^ & CD56^+^* | *CD69^-^/CD69^+^ 50/50* | *10.9* |
| *6* | *CD4^+^ ILC1* | *CD117^-^CRTH2^-^CD4^+^CD127^+^CD3^lo/-^* | *CD69^-^ 82%* | *0.86* |
| *7* | *CD161^-^ ILC3* | *CD45RA^+^CD161^-^CD127^+^CD117^+^CRTH2^-^NKp44^+^CD56^-^* | *CD69^-^ 74%* | *0.68* |
| *8* | *CD56^+^ LTi-like cells* | *NRP1^+^CD161^+^NKp44^+^CD117^+^CD45RA^-^ & CD56^+^* | *CD69^-^/CD69^+^ 50/50* | *12.6* |
| *9* | *CD161^-^ ILC1* | *CD45RA^+^CD161^-^CD127^+^CD117^-^CRTH2^-^NKp44^-^CD56^-^* | *CD69^-^ 86%* | *6.5* |
| *10* | *CD56^+^ LTi-like cells* | *NRP1^+^CD161^+^NKp44^+/-^CD117^+^CD45RA^-^ & CD56^+^* | *Resident/activated 90%* | *13.2* |
| *11* | *CD56^-^ naïve ILC3* | *CD45RA^+^CD25^+^CD161^+/-^CD62L^-/+^NKp44^-^CD117^+^ & CD56^-^* | *CD69^-^/CD69^+^ 50/50* | *14.4* |

**Supplementary Table 2.** Defining marker expression on ILC clusters from patients with small tonsils and moderate OSA. Clusters correspond to those depicted in Fig. 2 (A-D)

| **No.** | **Cluster** | **Phenotype** | **Tissue resident/activated (CD69^+^)** | **Frequency (%)** |
| --- | --- | --- | --- | --- |
| *1* | *CD62L^+^ naïve ILC3* | *CD45RA^+^CD62L^+^CD25^+^CD161^+/-^NKp44^-^CD117^+^ & CD56^+/-^* | *CD69^-^/CD69^+^*  *36/64* | *2.29* |
| *2* | *CD4^+^ ILC1* | *CD4^+^CD127^+^CD3^lo/-^* | *CD69^-^ 82%* | *2.86* |
| *3* | *CD56^+^ mature ILC3* | *NKp44^+^CD25^-^NRP1^-^CD161^+^CD45RA^+/-^CD62L^-^CD117^+^ & CD56^+^* | *Resident/activated 86%* | *13* |
| *4* | *CD56^-^ naïve ILC3* | *CD45RA^+^CD25^+^CD161^+/-^CD62L^-^NKp44^-^CD117^+^ & CD56^-^* | *CD69^-^/CD69^+^*  *70/30* | *13.2* |
| 5 | ILC2 | KLRG1^+^CD161^+^CD45RA^+/-^CD62L^-/+^CRTH2^+^CD25^+^ | *CD69^-^* 77% | 11 |
| *6* | *RA^-^ NKp44^-^ ILC3* | *NKp44^-^CD25^-/+^NRP1^-^CD161^+^CD45RA^-^CD62L^-^CD117^+^ & CD56^+/-^* | *CD69^-^/CD69^+^*  *50/50* | *3.33* |
| *7* | *CD56^+^ LTi* | *NRP1^+^CD161^+^NKp44^+^CD117^+^CD45RA^-^ & CD56^+^* | *Resident/activated*  *84%* | *18.5* |
| *8* | *CD56^+^ naïve ILC3* | *CD45RA^+^CD25^+^CD161^+/-^CD62L^-^NKp44^-^CD117^+^ & CD56^+^* | *CD69^-^/CD69^+^*  *50/50* | *14.7* |
| *9* | *CD161^-^ ILC1* | *CD45RA^+^CD161^-^CD127^+^CD117^-^CRTH2^-^NKp44^-^CD56^-^* | *CD69^-^ 86%* | *7.41* |
| *10* | *CD161^-^ ILC3* | *CD45RA^+^CD62L^-/+^CD161^-^CD127^+^CD117^+^CRTH2^-^NKp44^+/-^CD56^-^* | *CD69^-^* | *0.76* |
| *11* | *CD56^-^ LTi* | *NRP1^+^CD161^+^NKp44^+^CD117^+^CD45RA^-^ & CD56^-^* | *Resident/activated 80%* | *12.2* |
| *12* | *CD161^-^ ILC1* | *CD45RA^+^CD161^-^CD127^+^CD117^-^CRTH2^-^NKp44^+^CD56^+^* | *Resident/activated* | *0.79* |

**Supplementary Table 3.** Defining marker expression on ILC clusters from patients with large tonsils and very severe OSA. Clusters correspond to those depicted in Fig. 2 (E-H)

| **Antigen** | **Clone** | **Fluorophore** | **Manufacturer** |
| --- | --- | --- | --- |
| CD19 | 4G7 | FITC | BD |
| DCM | - | Far red | ThermoFisher |
| **CCR7** | G043H7 | APC-Cy7 | Biolegend |
| **ICOS** | C398.4A | v450 | Biolegend |
| **CD4** | RPA-T4 | BV510 | Biolegend |
| IgM | MHM-88 | BV570 | Biolegend |
| **PD1** | EH12.1 | BV605 | BD |
| **CD25** | BC96 | BV650 | Biolegend |
| **CD8** | RPA-T8 | BV711 | Biolegend |
| **CD3** | OKT3 | BV785 | Biolegend |
| **CD27** | O323 | PE | Biolegend |
| **CXCR5** | REA103 | PE-Vio615 | Miltenyi Biotec |
| CD20 | 2H7 | PE-Cy5 | Biolegend |
| **CD38** | HIT2 | PE-Cy7 | Biolegend |
| CD45 | HI30 | BUV395 | BD |
| **CD45RA** | HI100 | BUV737 | BD |

**Supplementary Table 4.** Staining panel used to identify T-cell and B-cell sub-populations in small and large tonsils. Markers, which are bold and underlined, were used to run UMAP and PhenoGraph algorithms on CD3^+^ T cells and CD19^+^CD20^+^ B cells, respectively.

| **No.** | **Cluster** | **Phenotype** | **Frequency (%)** |
| --- | --- | --- | --- |
| *1* | *CD38^-^ double negative* | *CD4^-^CD8^-^CD45RA^+/-^CCR7^-^ICOS^-^PD1^-^CD25^-^CXCR5^-^CD38^-^CD27^+^* | *2.1* |
| *2* | *Naïve CD8^+^* | *CD4^-^CD8^+^CD45RA^+^CCR7^+^ICOS^-^PD1^-^CD25^-^CXCR5^-^CD38^-^CD27^++^* | *8.8* |
| 3 | *CD25^lo^ T_FH_ CD4^+^* | CD4^+^CD8^-^CD45RA^-^CCR7^-^ICOS^++^PD1^+++^CD25^lo^CXCR5^+^CD38^+^CD27^++^ | 10.8 |
| *4* | *CD25^-^ T_FH_ CD4^+^* | *CD4^+^CD8^-^CD45RA^-^CCR7^-^ICOS^+^PD1^++^CD25^-^CXCR5^+^CD38^lo^CD27^++^* | *12.5* |
| *5* | *Double positive T_FH_-like* | *CD4^+^CD8^+^CD45RA^-^CCR7^-^ICOS^+^PD1^++^CD25^-/lo^CXCR5^+/-^  (75% positive) CD38^+^CD27^++^* | *2.6* |
| *6* | *Effector (Memory + TEMRA) CD8^+^* | *CD4^-^CD8^+^CD45RA^+/-^CCR7^-^ICOS^-^PD1^lo^CD25^-^CXCR5^-^CD38^-^CD27^++^* | *6.6* |
| *7* | *CCR7^-^ memory CD4^+^* | *CD4^+^CD8^-^CD45RA^-^CCR7^-/+^ICOS^++^PD1^+^CD25^-/lo^CXCR5^-^CD38^+^CD27^-/+^* | *5.2* |
| *8* | *CD25^+^ memory CD4^+^* | *CD4^+^CD8^-^CD45RA^-^CCR7^-/+^ICOS^++^PD1^+^CD25^+^CXCR5^-^CD38^-^CD27^++^* | *1.7* |
| *9* | *CD25^lo^ T_FH_ CD4^+^* | *CD4^+^CD8^-^CD45RA^-^CCR7^-^ICOS^++^PD1^+++^CD25^lo^CXCR5^++^CD38^+^CD27^+++^* | *5.9* |
| *10* | *Naïve CD4^+^* | *CD4^+^CD8^-^CD45RA^+^CCR7^+^ICOS^-^PD1^-^CD25^-^CXCR5^-^CD38^+/-^CD27^+^* | *15.5* |
| *11* | *CCR7^lo^ memory CD4^+^* | *CD4^+^CD8^-^CD45RA^-^CCR7^lo^ICOS^lo^PD1^-/lo^CD25^-^CXCR5^-^CD38^-^CD27^+^* | *16.7* |
| *12* | *CD38^+^ double negative* | *CD4^-^CD8^-^CD45RA^+^CCR7^-^ICOS^-^PD1^-^CD25^-^CXCR5^-^CD38^+^CD27^+++^* | *3.2* |
| *13* | *CXCR5^lo^ T_FH_ CD4^+^* | *CD4^+^CD8^-^CD45RA^-^CCR7^-/+^ ICOS^+^PD1^+^CD25^lo^CXCR5^lo^CD38^+^CD27^++^* | *8.5* |

**Supplementary Table 5.** Defining marker expression on T-cell clusters from patients with small tonsils and moderate OSA. Clusters correspond to those depicted in Fig. 3 (D-G)

| **No.** | **Cluster** | **Phenotype** | **Frequency (%)** |
| --- | --- | --- | --- |
| *1* | *CD45RA^-^ double negative* | *CD4^-^CD8^-^CD45RA^-^CCR7^-^ICOS^-^PD1^-^CD25^-^CXCR5^-^CD38^-^CD27^++^* | *0.52* |
| *2* | *CD27^-^ memory CD4^+^* | *CD4^+^CD8^-^CD45RA^-^CCR7^-^ICOS^+^PD1^lo^CD25^-/lo^CXCR5^-^CD38^-/lo^CD27^-^* | *0.54* |
| *3* | *CD25^lo^ T_FH_ CD4^+^* | *CD4^+^CD8^-^CD45RA^-^CCR7^-^ICOS^++^PD1^+++^CD25^lo^CXCR5^++^CD38^+^CD27^++^* | *17.2* |
| *4* | *CD27^+^ memory CD4^+^* | *CD4^+^CD8^-^CD45RA^-^CCR7^-^ICOS^+^PD1^+^CD25^+/-^CXCR5^-/lo^CD38^+^CD27^+^* | *9.95* |
| *5* | *Memory CD4^+^* | *CD4^+^CD8^-^CD45RA^-^CCR7^-/+^ICOS^lo^PD1^lo^CD25^-^CXCR5^-/lo^CD38^-/lo^CD27^+^* | *11.3* |
| *6* | *Naïve CD8^+^* | *CD4^-^CD8^+^CD45RA^+^CCR7^+^ICOS^-^PD1^-^CD25^-^CXCR5^-^CD38^-^CD27^+^* | *7.05* |
| *7* | *CCR7^+^ memory CD4^+^* | *CD4^+^CD8^-^CD45RA^-^CCR7^+^ICOS^+^PD1^+^CD25^lo^CXCR5^lo^CD38^+^CD27^++^* | *2.75* |
| *8* | *CD25^-^ T_FH_ CD4^+^* | *CD4^+^CD8^-^CD45RA^-^CCR7^-^ICOS^++^PD1^++^CD25^-^CXCR5^+^CD38^lo^CD27^++^* | *10.4* |
| *9* | *CCR7^-^ memory CD4^+^* | *CD4^+^CD8^-^CD45RA^-^CCR7^-^ICOS^lo^PD1^lo^CD25^-/+^CXCR5^-^CD38^-^CD27^++^* | *2.73* |
| *10* | *Double positive T_FH_-like* | *CD4^+^CD8^+^CD45RA^-^CCR7^-^ICOS^++^PD1^++^CD25^-/lo^CXCR5^+/-^  (65% positive) CD38^+^CD27^+^* | *2.72* |
| *11* | *CD45RA^+^ double negative* | *CD4^-^CD8^-^CD45RA^+^CCR7^-^ICOS^-^PD1^-^CD25^-^CXCR5^-^CD38^lo^CD27^+++^* | *3.52* |
| *12* | *Naïve CD4^+^* | *CD4^+^CD8^-^CD45RA^+^CCR7^+^ICOS^-^PD1^-^CD25^-^CXCR5^-^CD38^-/+^CD27^+^* | *15.4* |
| *13* | *Memory CD8^+^* | *CD4^-^CD8^+^CD45RA^-^CCR7^-^ICOS^-/lo^PD1^int^CD25^-^CXCR5^-^CD38^-/+^CD27^+^* | *5.72* |
| *14* | *TEMRA CD8^+^* | *CD4^-^CD8^+^CD45RA^+^CCR7^-^ICOS^-^PD1^int/-^CD25^-^CXCR5^-^CD38^-^CD27^++^* | *2.36* |
| *15* | *CXCR5^lo^ T_FH_ CD4^+^* | *CD4^+^CD8^-^CD45RA^-^CCR7^-^ICOS^++^PD1^+++^CD25^lo^CXCR5^lo^CD38^+^CD27^++^* | *7.86* |

**Supplementary Table 6.** Defining marker expression on T-cell clusters from patients with large tonsils and very severe OSA. Clusters correspond to those depicted in Fig. 3 (H-K).

| **No.** | **Cluster** | **Phenotype** | **Frequency (%)** |
| --- | --- | --- | --- |
| *1* | *Naïve B cells* | *CD19^+^CD20^+^IgM^lo^CD45RA^+^CD27^-^CXCR5^+^CD38^lo^* | *9.7* |
| *2* | *Plasma*  *blasts* | *CD19^++^CD20^lo^IgM^-^CD45RA^+/-^CD27^+++^CXCR5^-/lo^CD38^+++^* | *2.9* |
| *3* | *GC B cells* | *CD19^++^CD20^+++^IgM^-^CD45RA^+^CD27^+^CXCR5^+^CD38^++^* | *9.7* |
| *4* | *Naïve B cells* | *CD19^+^CD20^+^IgM^++^CD45RA^+^CD27^-^CXCR5^+^CD38^+^* | *10.6* |
| *5* | *Memory IgM* | *CD19^+^CD20^+^IgM^++^CD45RA^+^CD27^+^CXCR5^+^CD38^-^* | *6.4* |
| *6* | *CD38^+^ memory IgM* | *CD19^+^CD20^++^IgM^+^CD45RA^+^CD27^+^CXCR5^+^CD38^++^* | *2.98* |
| *7* | *Naïve B cells* | *CD19^+^CD20^lo^IgM^+^CD45RA^+^CD27^-^CXCR5^lo^CD38^-^* | *8* |
| *8* | *GC B cells* | *CD19^+^CD20^++^IgM^-^CD45RA^+^CD27^-/+^CXCR5^+^CD38^++^* | *7.6* |
| *9* | *GC B cells* | *CD19^lo^CD20^++^IgM^-^CD45RA^+^CD27^-^CXCR5^lo^CD38^+^* | *6.2* |
| *10* | *Naïve B cells* | *CD19^+^CD20^+^IgM^+^CD45RA^+^CD27^-^CXCR5^+^CD38^-^* | *9* |
| *11* | *CD27^-^ memory B cells* | *CD19^++^CD20^+^IgM^-^CD45RA^+^CD27^-^CXCR5^lo^CD38^-^* | *3.4* |
| *12* | *Naïve B cells* | *CD19^+^CD20^+^IgM^++^CD45RA^+^CD27^-^CXCR5^+^CD38^lo^* | *9.8* |
| *13* | *Memory B cells* | *CD19^+^CD20^+^IgM^-^CD45RA^+^CD27^+^CXCR5^+^CD38^-^* | *13.6* |

**Supplementary Table 7.** Defining marker expression on B-cell clusters from patients with small tonsils and moderate OSA. Clusters correspond to those depicted in Fig. 4 (B-E).

| **No.** | **Cluster** | **Phenotype** | **Frequency (%)** |
| --- | --- | --- | --- |
| 1 | *GC B cells* | *CD19^+^CD20^+++^IgM^-^CD45RA^+^CD27^-^CXCR5^+^CD38^++^* | *5.28* |
| 2 | *CD27^-^ memory B cells* | *CD19^++^CD20^++^IgM^-^CD45RA^+^CD27^-^CXCR5^-/lo^CD38^-^* | *2.09* |
| 3 | *Naïve B cells* | *CD19^+^CD20^+^IgM^++^CD45RA^+^CD27^-^CXCR5^+^CD38^lo^* | *7.5* |
| 4 | *GC B cells* | *CD19^++^CD20^+++^IgM^-^CD45RA^+^CD27^+^CXCR5^+^CD38^++^* | *7.05* |
| 5 | *Naïve B cells* | *CD19^+^CD20^+^IgM^+^CD45RA^+^CD27^-^CXCR5^+^CD38^-^* | *8.96* |
| 6 | *Naïve B cells* | *CD19^++^CD20^++^IgM^++^CD45RA^+^CD27^-^CXCR5^+^CD38^-^* | *2.57* |
| 7 | *Plasma blasts* | *CD19^++^CD20^lo^IgM^-^CD45RA^+/-^CD27^+++^CXCR5^-/lo^CD38^+++^* | *1.69* |
| 8 | *CD38^+^ memory IgM* | *CD19^+^CD20^++^IgM^++^CD45RA^+^CD27^+^CXCR5^+^CD38^++^* | *4.99* |
| 9 | *Memory B cells* | *CD19^+^CD20^+^IgM^-^CD45RA^+^CD27^+^CXCR5^lo^CD38^-^* | *7.83* |
| 10 | *Naïve B cells* | *CD19^+^CD20^+^IgM^lo^CD45RA^+^CD27^-^CXCR5^+^CD38^lo^* | *4.77* |
| 11 | *Naïve B cells* | *CD19^+^CD20^+^IgM^+^CD45RA^+^CD27^-^CXCR5^+^CD38^-^* | *3.46* |
| 12 | *Naïve B cells* | *CD19^+^CD20^+^IgM^++^CD45RA^+^CD27^-^CXCR5^+^CD38^lo^* | *12.2* |
| 13 | *Naïve B cells* | *CD19^+^CD20^+^IgM^+^CD45RA^+^CD27^-^CXCR5^+^CD38^lo^* | *9.45* |
| 14 | *Naïve B cells* | *CD19^+^CD20^+^IgM^lo^CD45RA^+^CD27^-^CXCR5^+^CD38^-^* | *10.6* |
| 15 | *GC B cells* | *CD19^lo^CD20^++^IgM^-^CD45RA^+^CD27^-^CXCR5^lo^CD38^+^* | *6.47* |
| 16 | *Memory IgM* | *CD19^+^CD20^+^IgM^++^CD45RA^+^CD27^+^CXCR5^+^CD38^-^* | *5.08* |

**Supplementary Table 8.** Defining marker expression on B-cell clusters from patients with large tonsils and very severe OSA. Clusters correspond to those depicted in Fig. 4 (F-I).

| **Antigen** | **Clone** | **Fluorophore** | **Manufacturer** |
| --- | --- | --- | --- |
| CD11c | 3.9 | FITC | Biolegend |
| Ki67 | B56 | A700 | BD |
| DCM |  | nearIR | ThermoFisher |
| CD20 | 2H7 | PacificBlue | Biolegend |
| CD45 | HI30 | v500 | BD |
| CD21 | B-ly4 | BV605 | BD |
| CD3 | SK7 | BV711 | Biolegend |
| CD19 | HIB19 | BV786 | BD |
| CD27 | O323 | PE | Biolegend |
| T-bet | O4-46 | PE-CF594 | BD |
| CD38 | HIT2 | PE-Cy7 | Biolegend |
| FcRL4 | A1 | BUV395 | BD |
| FcRL5 | 509F6 | BUV737 | BD |

**Supplementary Table 9.** Staining panel used to identify atypical memory B-cell sub-populations in small and large tonsils. Markers used to run UMAP and PhenoGraph algorithms on CD19^+^CD20^+^ B cells are underlined.

| **No.** | **Cluster** | **Phenotype** | **Frequency (%)** |
| --- | --- | --- | --- |
| *1* | *CD27^-^ B cells* | *CD11c^-^KI67^-^FcRL4^-^FcRL5^-^CD20^+^ CD21^lo/+^CD27^-^T-bet^-^CD38^lo^* | *6.98* |
| *2* | *CD27^-^ B cells* | *CD11c^-^KI67^-/+^FcRL4^-^FcRL5^-^CD20^lo^ CD21^-/lo^CD27^-/+^T-bet^-^CD38^lo^* | *5.32* |
| *3* | *CD27^+^ B cells* | *CD11c^-^KI67^-^FcRL4^-^FcRL5^-^CD20^++^ CD21^+^CD27^+^T-bet^-^CD38^-/lo^* | *13.6* |
| *4* | *CD27^-^ B cells* | *CD11c^-^KI67^-^FcRL4^-^FcRL5^-^CD20^+ +^CD21^++^CD27^-^T-bet^-^CD38^lo^* | *14.9* |
| *5* | *Plasma blasts* | *CD11c^-^KI67^-^FcRL4^-^FcRL5^+^CD20^lo^ CD21^+^CD27^+++^T-bet^-^CD38^+++^* | *4.57* |
| *6* | *GC B cells* | *CD11c^-^KI67^++^FcRL4^-^FcRL5^-^CD20^+++^ CD21^++^CD27^+^T-bet^-^CD38^+^* | *9.8* |
| *7* | *CD27^-^ B cells* | *CD11c^-^KI67^-^FcRL4^-^FcRL5^-^CD20^+^ CD21^lo/+^CD27^-^T-bet^-^CD38^-^* | *10.6* |
| *8* | *GC B cells* | *CD11c^-^KI67^+^FcRL4^-^FcRL5^-^CD20^+++^ CD21^+^CD27^-/+^T-bet^-^CD38^+^* | *9.43* |
| *9* | *CD27^-^ B cells* | *CD11c^-^KI67^-^FcRL4^-^FcRL5^-^CD20^+^CD21^lo^CD27^-^T-bet^-^CD38^+^* | *2.07* |
| *10* | *CD27^-^ B cells* | *CD11c^-^KI67^-^FcRL4^-^FcRL5^-^CD20^+^CD21^lo/+^CD27^-^T-bet^-^CD38^lo^* | *12.6* |
| *11* | *Atypical B cells* | *CD11c^lo^KI67^-/+^ (58/42%) FcRL4^+^FcRL5^+^CD20^+++^CD21^-/+^CD27^-/+^T-bet^-/+^ (70/30%) CD38^-/lo^* | *10.1* |

**Supplementary Table 10.** Defining marker expression on B-cell clusters from patients with small tonsils and moderate OSA (atypical B cells). Clusters correspond to those depicted in Fig. 5 (A-D).

| **No.** | **Cluster** | **Phenotype** | **Frequency (%)** |
| --- | --- | --- | --- |
| *1* | *CD27^-^ B cells* | *CD11c^-^KI67^-^FcRL4^-^FcRL5^-^CD20^+^ CD21^lo/+^CD27^-^T-bet^-^CD38^-^* | *6.95* |
| *2* | *GC B cells* | *CD11c^-^KI67^++^FcRL4^-^FcRL5^-^CD20^+++^ CD21^+^CD27^+^T-bet^-^CD38^+^* | *8.84* |
| *3* | *CD27^-^ Atypical B cells* | *CD11c^lo^KI67^-/+^(30/70%)FcRL4^+^FcRL5^-/+^CD20^+++^CD21^-/+^CD27^-^T-bet^-/+^ (75/25%)CD38^-/lo^* | *7.5* |
| *4* | *CD27^-^ B cells* | *CD11c^-^KI67^-^FcRL4^-^FcRL5^-^CD20^+^ CD21^+^CD27^-^T-bet^-^CD38^lo^* | *9.98* |
| *5* | *CD27^-^ B cells* | *CD11c^-^KI67^-^FcRL4^-^FcRL5^-^CD20^+^ CD21^++^CD27^-^T-bet^-^CD38^lo^* | *11.2* |
| *6* | *CD27^-^ B cells* | *CD11c^-^KI67^-/+^FcRL4^-^FcRL5^-^CD20^lo^ CD21^lo^CD27^-^T-bet^-^CD38^lo^* | *5.19* |
| *7* | *Plasma blasts* | *CD11c^-^KI67^-^FcRL4^-^FcRL5^+^CD20^lo^ CD21^+^CD27^++^T-bet^-^CD38^+++^* | *3.08* |
| *8* | *CD27^-^ B cells* | *CD11c^-^KI67^-^FcRL4^-^FcRL5^-^CD20^+^ CD21^+^CD27^-^T-bet^-^CD38^-/lo^* | *10.2* |
| *9* | *CD27^+^ Atypical B cells* | *CD11c^lo^KI67^-/+^(82/18%)FcRL4^+^FcRL5^+^CD20^+++^CD21^lo^CD27^+^T-bet^-/+^(75/25%)CD38^-^* | *1.58* |
| *10* | *GC B cells* | *CD11c^-^KI67^+^FcRL4^-^FcRL5^-^CD20^+++^ CD21^++^CD27^-/+^T-bet^-^CD38^+^* | *10.5* |
| *11* | *CD27^-^ B cells* | *CD11c^-^KI67^-^FcRL4^-^FcRL5^-^CD20^+^ CD21^++^CD27^-^T-bet^-^CD38^-/lo^* | *6.33* |
| *12* | *CD27^-^ B cells* | *CD11c^-^KI67^-^FcRL4^-^FcRL5^-/+^CD20^+^ CD21^++^CD27^-^T-bet^-^CD38^-^* | *8.87* |
| *13* | *CD27^+^ B cells* | *CD11c^-^KI67^-^FcRL4^-^FcRL5^-^CD20^++^ CD21^+^CD27^+^T-bet^-^CD38^-^* | *9.81* |

**Supplementary Table 11.** Defining marker expression on B-cell clusters from patients with large tonsils and very severe OSA (atypical B cells). Clusters correspond to those depicted in Fig. 5 (E-H).

# Supplementary Figures

**
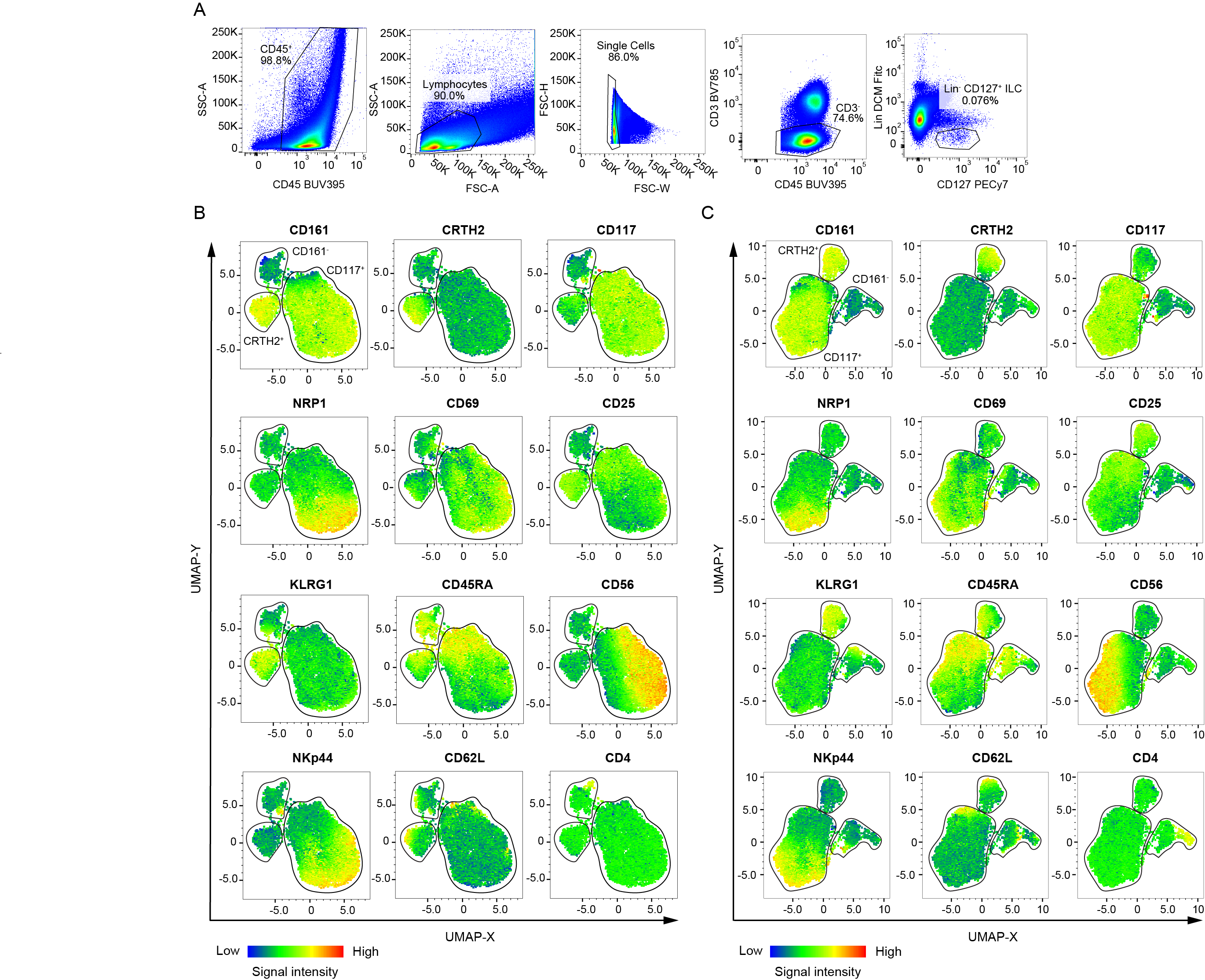
**

**Supplementary Figure 1.** **(A)** Gating strategy for CD127^+^ ILCs. **(B, C)** UMAP of CD127^+^ ILCs in small **(B)** and large **(C)** tonsils. Major UMAP groups of CD117^+^, CRTH2^+^, as well as CD161^-^ ILCs are outlined with borders.

**
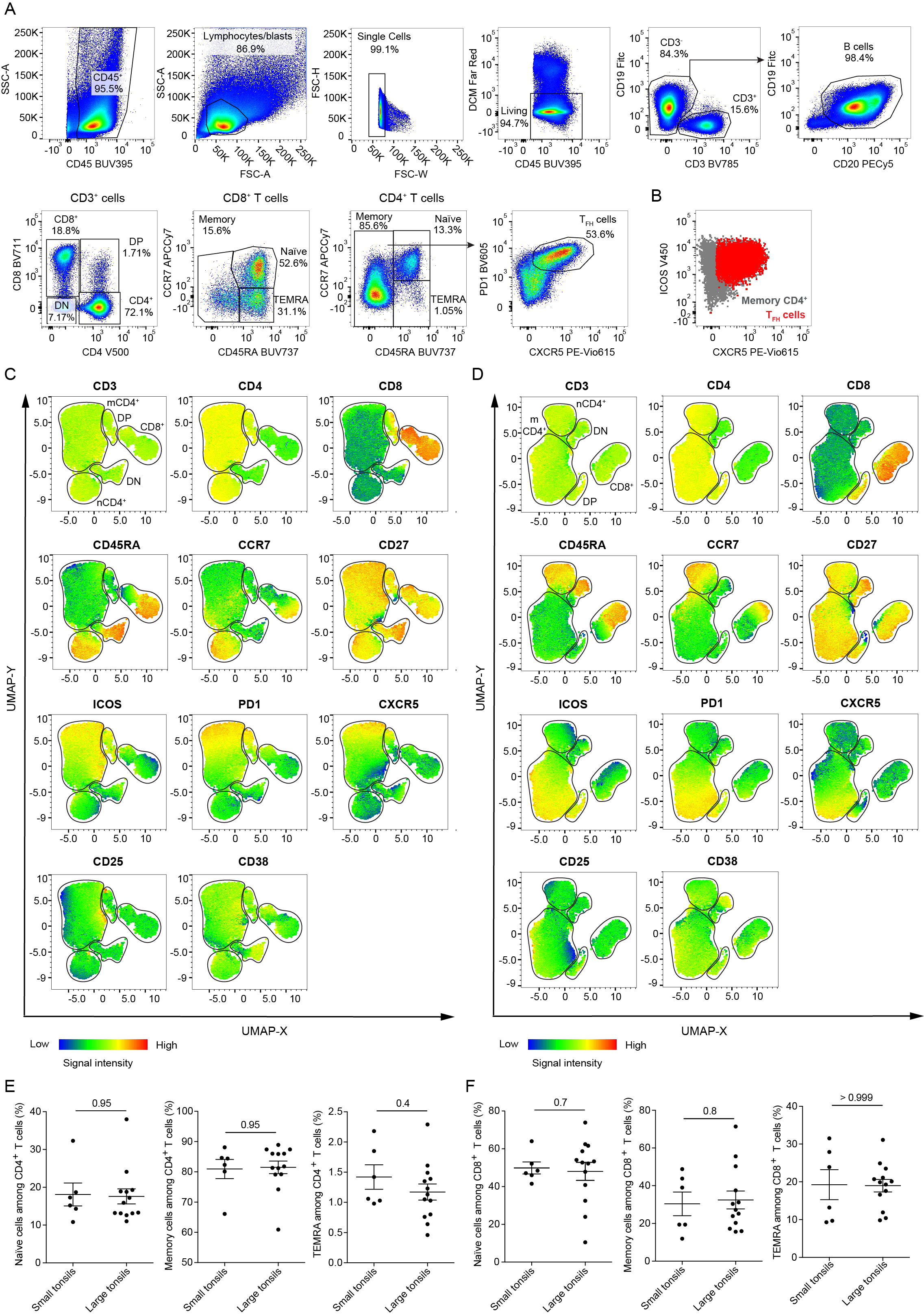
**

**Supplementary Figure 2. (A)** Gating strategy for B cells and T cells, as well as naïve, TEMRA and memory T-cell subsets and T_FH_ cells. **(B)** Representative plot of ICOS vs. CXCR5 expression on memory CD4^+^ T cells and T_FH_ cells. **(C, D)** UMAP of CD3^+^ T cells in small **(C)** and large **(D)** tonsils. UMAP groups of memory CD4^+^ (mCD4^+^), naïve CD4^+^ (nCD4^+^), CD8^+^, double positive (DP) and double negative (DN) T cells and outlined with borders **(E, F)** Frequencies of naïve, memory and TEMRA CD4^+^ **(E)** and CD8^+^ **(F)** T cells in small and large tonsils. Lines represent mean ± SEM. Statistical significance was calculated using Mann-Whitney *U* test.


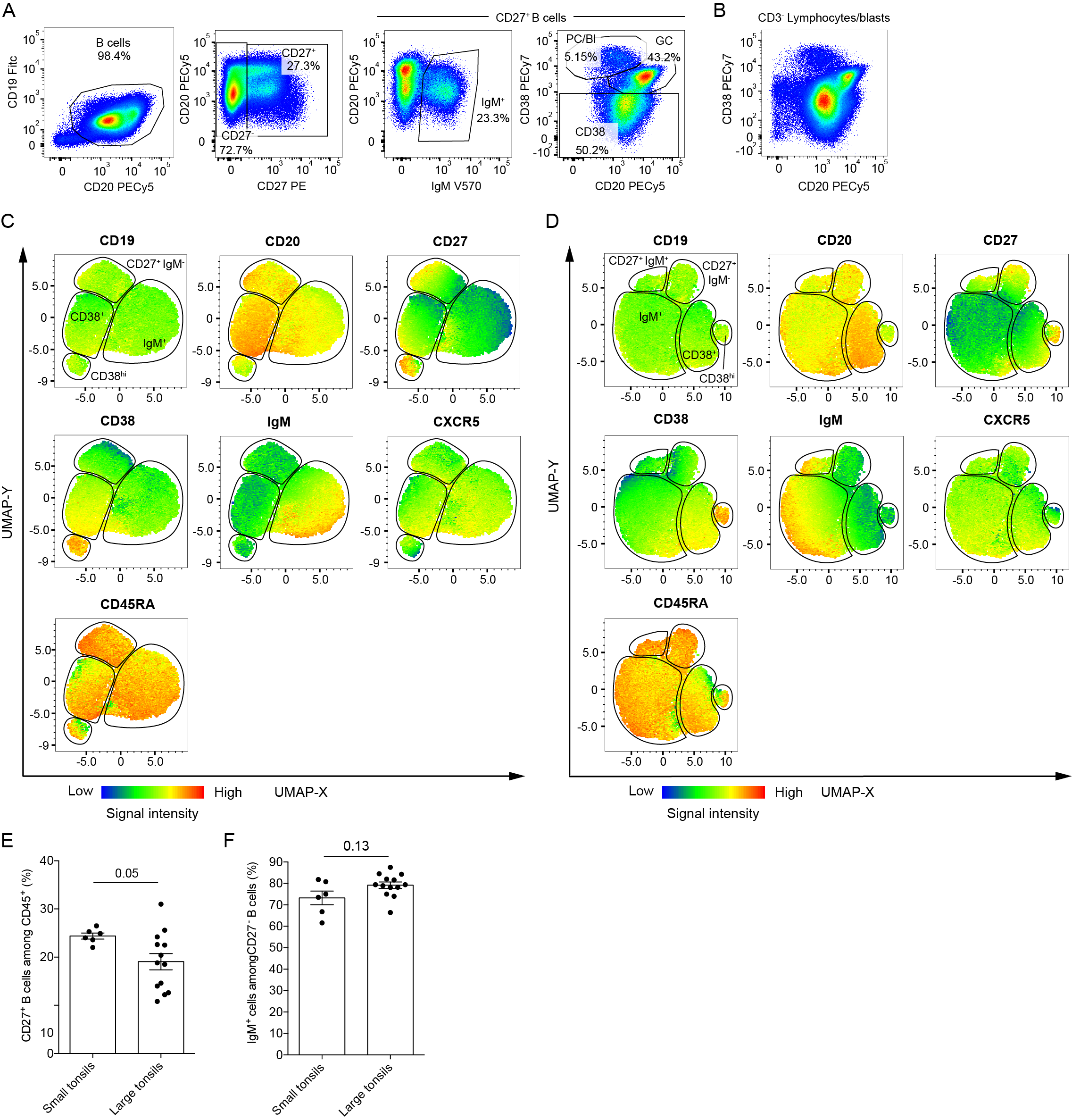


**Supplementary Figure 3. (A)** Gating strategy for assessing B-cell sub-populations among B cells gated as in supplementary figure 2A. **(B)** Representative plot of CD38 vs. CD20 expression on total living CD3^-^ lymphocytes and lymphoblasts. **(C, D)** UMAP of CD19^+^CD20^+^ B cells in small **(C)** and large **(D)** tonsils. UMAP groups of CD38^hi^, CD38^+^, IgM^+^, CD27^+^IgM^-^ and CD27^+^IgM^+^ B cells are outlined with borders. **(E)** Frequency of CD27^+^ B cells among total living CD45^+^ cells in small and large tonsils. **(F)** Frequency of IgM^+^ cells among CD27^-^ B cells in small and large tonsils. **(E, F)** Bars and error bars represent mean ± SEM. Statistical significance was calculated using Mann-Whitney *U* test.


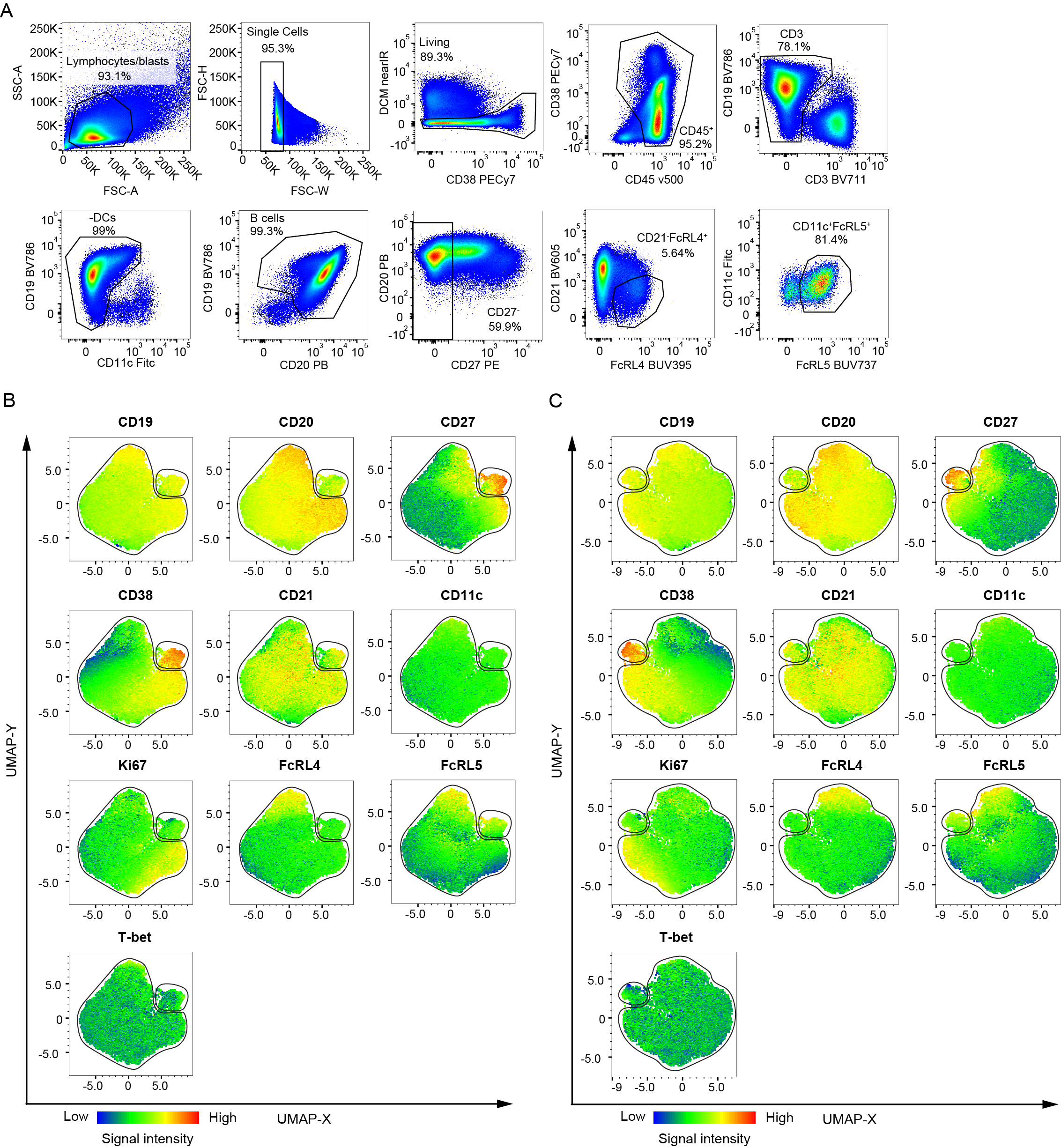


**Supplementary Fig. 4 (A)** Gating strategy for B cells and atypical memory B cells. **(B, C)** UMAP of CD19^+^CD20^+^ B cells in small **(B)** and large **(C)** tonsils. Identified UMAP groups are outlined with borders.
